# Network and synaptic mechanisms underlying high frequency oscillations in the rat and cat olfactory bulb under ketamine-xylazine anesthesia

**DOI:** 10.1038/s41598-021-85705-5

**Published:** 2021-03-18

**Authors:** Władysław Średniawa, Jacek Wróbel, Ewa Kublik, Daniel Krzysztof Wójcik, Miles Adrian Whittington, Mark Jeremy Hunt

**Affiliations:** 1 Nencki Institute of Experimental Biology of Polish Academy of Sciences, 3 Pasteur Street, 02-093 Warsaw, Poland; 2 University of Warsaw, Faculty of Biology, Miecznikowa 1, 02-096 Warsaw, Poland; 3 Faculty of Management and Social Communication, Jagiellonian University, 30-348 Cracow, Poland; 4 University of York, Heslington, NY YO10 5DD United Kingdom

**Keywords:** Physiology, Neuroscience, Neuronal physiology, Olfactory system, Synaptic transmission, Experimental models of disease, Translational research, Psychiatric disorders, Psychosis, Schizophrenia

## Abstract

Wake-related ketamine-dependent high frequency oscillations (HFO) can be recorded in local field potentials (LFP) from cortical and subcortical regions in rodents. The mechanisms underlying their generation and occurrence in higher mammals are unclear. Unfortunately, anesthetic doses of pure ketamine attenuate HFO, which has precluded their investigation under anesthesia. Here, we show ketamine-xylazine (KX) anesthesia is associated with a prominent 80–130 Hz rhythm in the olfactory bulb (OB) of rats, whereas 30–65 Hz gamma power is diminished. Simultaneous LFP and thermocouple recordings revealed the 80–130 Hz rhythm was dependent on nasal respiration. This rhythm persisted despite surgical excision of the piriform cortex. Silicon probes spanning the dorsoventral aspect of the OB revealed this rhythm was strongest in ventral areas and associated with microcurrent sources about the mitral layer. Pharmacological microinfusion studies revealed dependency on excitatory-inhibitory synaptic activity, but not gap junctions. Finally, a similar rhythm occurred in the OB of KX-anesthetized cats, which shared key features with our rodent studies. We conclude that the activity we report here is driven by nasal airflow, local excitatory-inhibitory interactions, and conserved in higher mammals. Additionally, KX anesthesia is a convenient model to investigate further the mechanisms underlying wake-related ketamine-dependent HFO.

## Introduction

Local field potential (LFP) oscillations reflect synchronous activity of neuronal assemblies and are thought to play a crucial role in information processing^1,2^. Recent years have witnessed a surge of interest in high frequency oscillations (HFO, >100 Hz), also known as ripples, considered important for their roles in health and disease^3,4^. HFO have been investigated most notably in the rodent hippocampus, but are also found in diverse cortical, olfactory and limbic areas^5–7^, where they have been linked to near-death states^8^, seizures^9^, Parkinson’s disease^10^, and models of psychoses^11^. Collectively, these results point to regional and state dependent mechanisms underlying different types of HFO generation in the brain.

N-methyl-D-aspartate receptors (NMDAR) are expressed widely in the CNS, and are well-known to be involved in learning and memory processes^12^. It is well-documented, that, at subanesthetic doses, ketamine (and related NMDAR antagonists) increases the HFO power in many cortical and subcortical areas. These studies have been carried out almost exclusively in freely moving rodents, and have addressed several aspects including the neuroatomical profile, dose-dependency, cross frequency coupling, and relationships with overt behaviour^11,13–25^. We recently provided evidence that the olfactory bulb (OB) is a strong generator of HFO in rodents after a subanesthetic dose^26^. The OB appears to have a particularly privileged position since it can impose both fast and slow oscillatory activity in distant regions^27^. Indeed, bursts of ketamine-dependent HFO tightly couple to nasal respiration, and naris blockade reduces HFO power locally within the OB, and in the ventral striatum and prefrontal cortex^25^. However, there remain important gaps in our understanding, perhaps most notably, which networks and synaptic mechanisms underlie this type of HFO, and does a comparable rhythm occur in higher mammals.

Anesthetized states can provide distinct advantages when studying fundamental oscillatory networks in the OB, since core neural networks along with respiration are spared, whilst behavioural confounds are removed. Unfortunately, at anesthetic doses (150–200 mg/kg), ketamine attenuates HFO power in rats^11,28^ which is followed by rebound increases in HFO power associated with the recovery phase. Many other anesthetics (pentobarbital, isoflurane, urethane and chloral hydrate) also attenuate ketamine-dependent HFO^24,29^ but see^30^ for fentanyl, severely limiting the methods available to experimentally investigate this rhythm. Notably, when combined with the sedative, xylazine, ketamine can be administered at lower doses, and this mixture is routinely used for animal anesthesia. Although anesthesia generally reduces the power^31^, and frequency^32^ of fast oscillations, there is some evidence that HFO can be observed under ketamine-xylazine (KX)^33,34^. It is unknown if this activity is related to ketamine-dependent HFO recorded during waking, or to classical gamma activity. Given the above we developed the following workflow; we firstly tested the hypothesis that ketamine-dependent HFO and fast oscillations recorded under KX are related. We then probed the potential mechanisms of HFO rhythmogenesis, using a variety of experimental techniques, including simultaneous LFP/thermocouple recordings, current source density analyses, local pharmacological microinfusion studies, and surgical excision of major afferent input. Finally, to determine the potential translatablility of our findings to higher mammals we examined if a similar rhythm could be recorded in KX anesthetized cats.

## Results

### KX and subanesthetic ketamine both reduce gamma power (30–65 Hz) but increase the power of faster rhythms.

**Figure 1:**
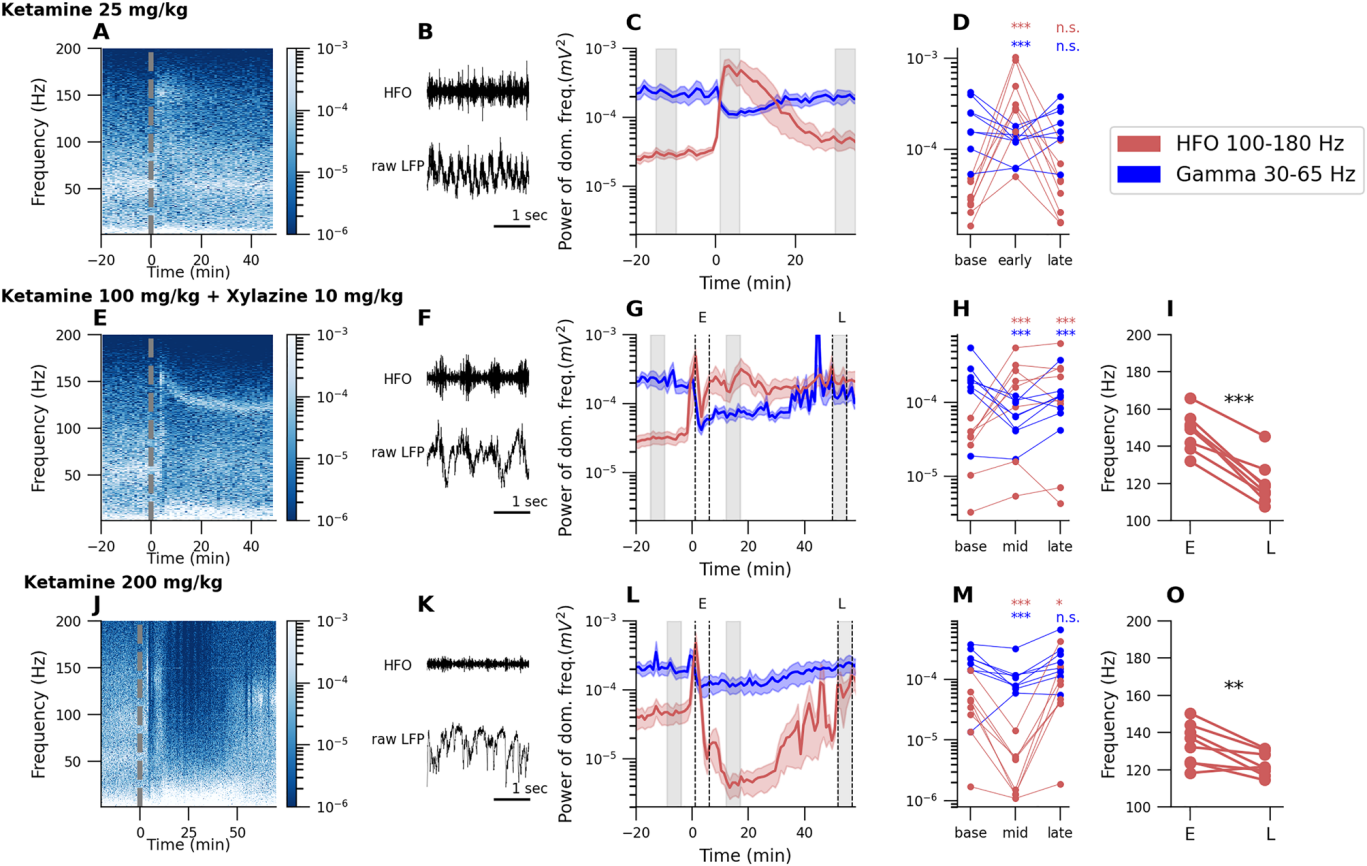
Effect of ketamine (subanesthetic and anesthetic doses) and KX anesthesia on 30–65 Hz and 100–180 Hz oscillations in the rat OB. First row (**A**–**D**) presents results for subanesthetic ketamine 25 mg/kg, second row (**E**–**I**) for ketamine 100 mg/kg + xylazine 10 mg/kg (KX) anesthesia and third row for anesthetic dose of ketamine 200 mg/kg. First column (**A**,**E**,**J**): Example spectrograms computed from the OB before and after injection. Dotted line at $t\,=\,0$ is injection time. Second column (**B**,**F**,**K**): Example raw waveform (bottom) and filtered signal in HFO band 100–180 Hz (top). Third column (**C**,**G**,**L**): Extracted data from spectrograms of the power of dominant frequency for 30–65 Hz and 100–180 Hz activity (mean across rats n = 8). The column (**D**,**H**,**M**): Individual traces of HFO and gamma power taken from three time points (marked with grey bars in **C**,**G**,**L** plots). Both subanesthetic ketamine and KX administration reduced gamma power and substantially increased HFO power (*p<0.05, **p<0.01, ***p<0.001, and n.s. (not significant) with respect to baseline). (**I**,**O**): Comparison of dominant frequency for the 100–180 Hz band just after injection of ketamine 25 mg/kg or KX for early E and late L time points (**p<0.01, ***p<0.001).

Subanesthetic ketamine (Fig. 1A–D): Intraperitoneal injection of ketamine (25 mg/kg) produced an immediate and substantial increase in HFO ($152.37 \pm 10.21$ Hz, p = 0.0015, Friedman test (F-stat = 13.0) with Nemenyi post-hoc: p<0.001, n = 8). We also observed a concomitant reduction in the power of classical gamma oscillations 30–65 Hz in LFPs recorded from the rodent OB (p = 0.044, Friedman test (F-stat = 6.25) with Nemenyi post-hoc test: p<0.001, n = 8). This is in line with reductions in gamma power reported after intra-OB infusion of NMDAR antagonists^35^.

KX anesthesia (Fig. 1E–I: Intraperitoneal injection (100 mg/kg ketamine + 10 mg/kg xylazine) produced an unconscious state confirmed by loss of tail-pinch reflex and eyeblink reflexes which lasted 30–40 min. This was also associated with an increase in the power of HFO almost immediately after injection which lasted for the duration of the experiment (p = 0.0098, Friedman test (F-stat = 9.25) with Nemenyi post-hoc: p<0.001 (both mid and late time points), n = 8). Over the course of an hour the fast rhythm gradually slowed in frequency to $119.96 \pm 11.37$ Hz (Fig. 1I, p<0.001, Student’s paired t-test, n = 8). Notably, HFO power under KX was significantly smaller compared to 25 mg/kg ketamine (mean HFO power after KX$\,=\,0.2 \times 10^{-3} \pm 0.15 \times 10^{-3} \mathrm{mV}^{2}$, mean HFO power after subanesthetic ketamine $\,=\,0.56 \times 10^{-3} \pm 0.34 \times 10^{-3} \mathrm{mV}^{2}$, p = 0.036, Wilcoxon test, n = 8). Consistent with the 25 mg/kg ketamine dose, KX anesthesia also produced a concomitant reduction in 30–65 Hz activity (p = 0.0022, Friedman test (F-stat = 12.25) with Nemenyi post-hoc: p<0.001 (both time points), n = 8).

Anesthetic ketamine (Fig. 1J–O): We also examined the effect of an anesthetic dose of ketamine on oscillatory activity recorded in the OB. We chose 200 mg/kg since pilot studies showed that this dose produce a depth of anesthesia comparable to KX. This was associated with a reduction in the power fast activity which lasted around 30 min, followed by rebound HFO associated with recovery of the righting reflex and waking (p = 0.0008, Friedman test (F-stat = 14.25) with Nemenyi post-hoc:, p<0.001 and p = 0.045, n = 8) consistent with^11,28^. Like KX, HFO that emerged during recovery were slower in frequency $122.975 \pm 8.86$ Hz (Fig. 1O, p = 0.0099, Student’s paired t-test, n = 8). Gamma power was also reduced when the rat was anesthetized (p = 0.03, Friedman test (F-stat = 7.0) with Nemenyi post-hoc:, p<0.001, n = 8). Attenuation of most EEG oscillatory activity (described as EEG holes) during ketamine anesthesia has also been reported recently in sheep^36^.

At the end of this study, four rats received an injection of 10 mg/kg xylazine which had no significant effect on 100−180 Hz activity or 30-65 Hz gamma (Fig. Supplementary S1 A and B). This indicated that ketamine rather than xylazine was responsible for the effect we observed.

### KX-dependent 80–130 Hz oscillations are entrained by nasal respiration.

**Figure 2:**
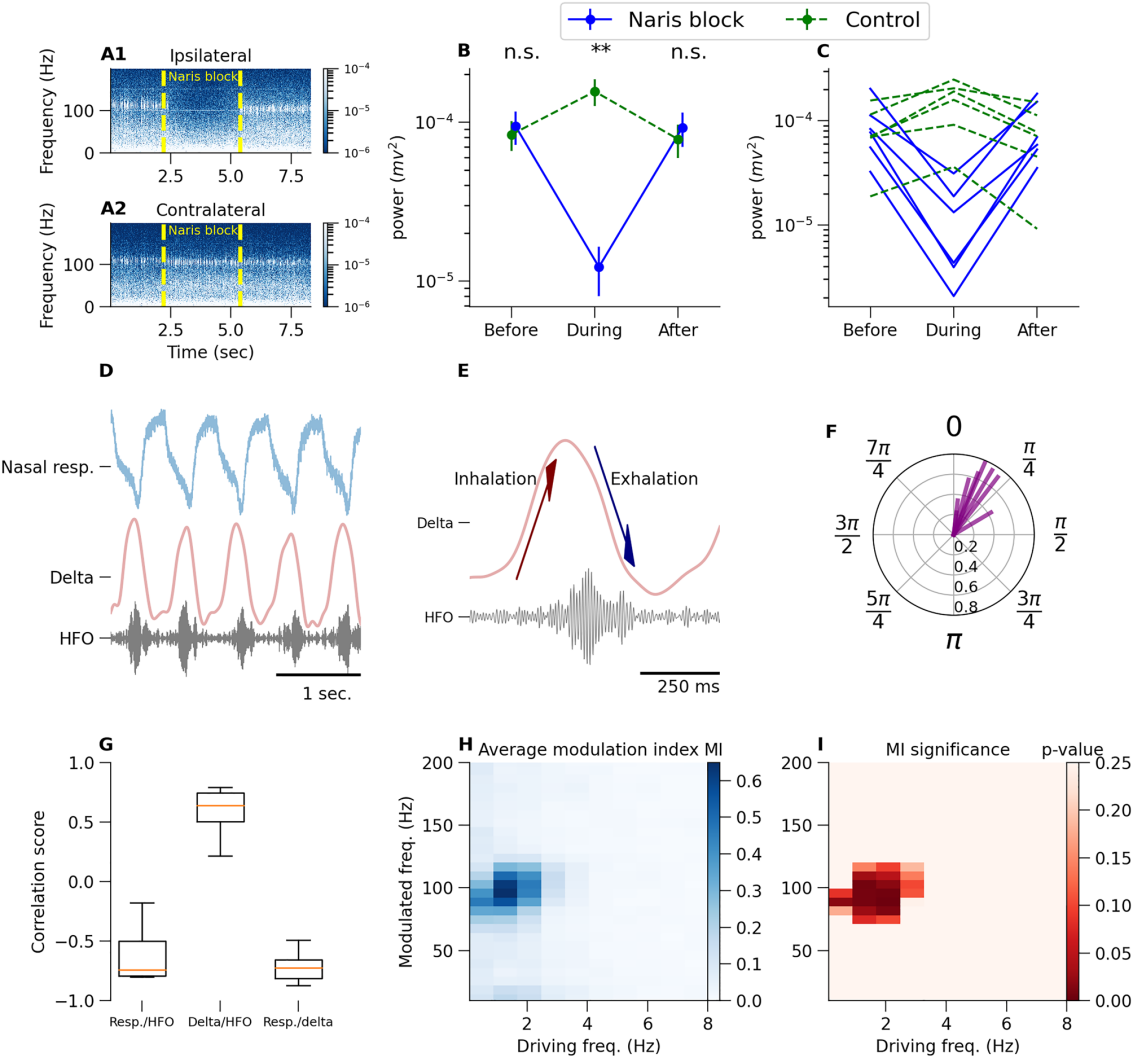
Nasal respiration modulates KX-dependent 80–130 Hz oscillations in the OB of anesthetized rats. (**A**) Spectrogram computed from ipsilateral and contralateral to the naris blockade OB channels. Dotted lines relate to naris blockade time. (**B**) Extracted data from spectrogram of power of dominant frequency in 80–130 Hz range (average across rats n = 8). Unilateral naris blockade reduced 80–130 Hz power on the ipsilateral side but increased 80–130 Hz power contralateral to blockade. Values from individual rats are shown in (**C**). (**D**) Simultaneous OB LFP and respiratory rhythm recorded using thermocouples. (**E**) Example recordings of simultaneous OB LFP for delta (0.3–3 Hz) and the 80–130 Hz rhythm. Arrows show respiratory cycle phase. (**F**) Phase plot of the peak of the burst of KX 80–130 Hz activity relative to delta peak (phase zero). Each radius represents ITPC (see "Methods") of one rat. (**G**) Waveform correlation scores calculated for 5 min for respiration rhythm and OB LFP delta (first box plot), delta and the envelope of 80–130 activity (second box plot), and respiration rhythm and OB LFP delta (third box plot). Box plots show first to third quartile of the correlation scores, median (orange line) and all data range of n = 8 rats (error bars). (**H**) Modulation index matrix (averaged across rats n = 8) for 5 min of continuous LFP signal under KX anesthesia. (**I**) Resampling (across rats) statistics for modulation index “pixels”. Control group for resampling were rats under isoflurane anesthesia (**p<0.01 and n.s. (not significant) with respect to control).

Slow oscillations in the mammalian OB have long been known to be closely related to nasal respiration, and we have recently found that in freely moving rats bursts of ketamine-dependent HFO are respiratory-related^25^. We investigated the relationship between nasal respiration and KX fast oscillations further using a thermocouple implanted in the naris of rats. These experiments, and the rat experiments described hereafter, were carried out after initial isoflurane anesthesia necessary for surgical procedures, which was then substituted for KX. As expected, shortly after injection of KX, fast rhythmic activity was visible in raw OB LFPs, nested on a slower oscillatory rhythm (Fig. 2D,E). Prior isoflurane exposure reduced the mean frequency of HFO compared to KX alone (Fig. Supplementary S2 B, p = 0.001, Student’s paired t-test, n = 6). For this reason, in these experiments the 80–130 Hz band was used. Although gamma oscillations in the OB can reach 80 Hz, considering that gamma power is largely attenuated under KX anesthesia, and since others shown that local injection of APV or MK801 reduces the power both low and high gamma in the OB^35^, we reasoned there would be little contamination of 80–130 Hz band by gamma.

Unilateral naris blockade immediately reduced the power of 80–130 Hz activity (Fig. 2A–C, p = 0.0013, one-way ANOVA, n = 6). The reduction of 80–130 Hz activity power in the OB occurred exclusively on the ipsilateral side and quickly recovered when blockade was removed. Interestingly, a small increase in 80–130 Hz activity power was recorded in the contralateral side.

Fast oscillations after subanesthetic ketamine and KX anesthesia occurred as discrete bursts, lasting around 100 ms, nested towards the peaks of slow frequencies (Fig. 1B,F). In rats injected with 25 mg/kg ketamine coupling occurs at theta frequencies^25,37^. Under KX, we also observed coupling, but this occurred at slower, delta frequencies; see Fig. 2D for an example recording of nasal respiration, and its relation to $< 3$ Hz, and 80–130 Hz activities recorded in the OB. Next, we computed correlation coefficients between nasal respiration, $< 3$ Hz filtered signal and the 80–130 Hz envelope (Fig. 2G). We found bursts temporally correlated with peaks of local delta and troughs of the thermocouple signal (Fig. 2D,G). Phase analyses (Fig. 2E,F) revealed that the peak of the envelope of fast oscillatory bursts occurred preferentially on the descending phase of delta oscillations (Fig. 2F). The peak phase of local slow LFP rhythm corresponds to inhalation-exhalation transition in breathing cycle (Fig. 2E). We also calculated the modulation index between the OB LFP and nasal respiration signals (Fig. 2H,I), see "Methods" for computational details, and confirmed that 80–130 Hz oscillations are driven by nasal respiration.

### KX-dependent 80–130 Hz oscillations are associated with dipole-like current sources around the mitral cell layer.

**Figure 3:**
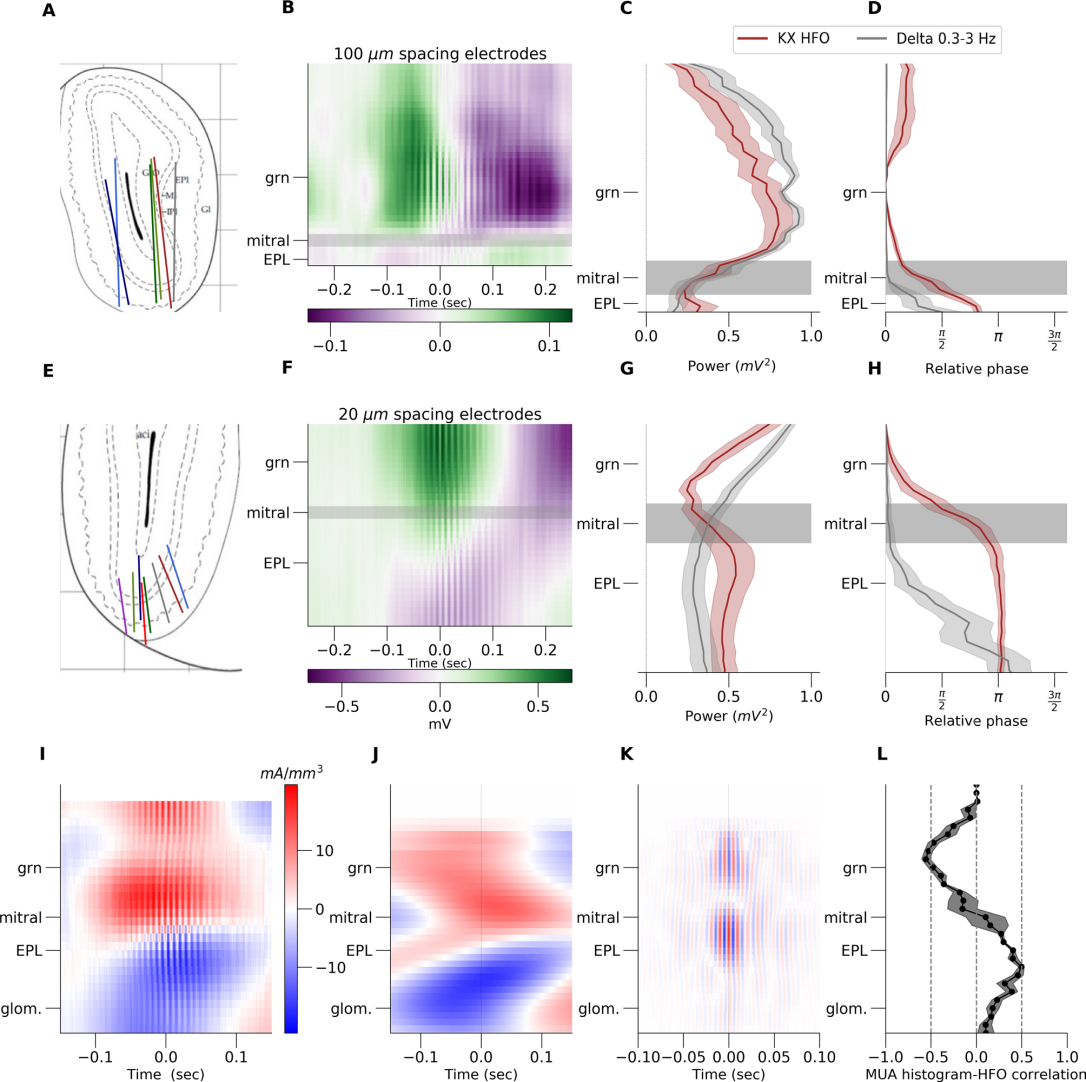
Depth profile of KX-dependent 80–130 Hz oscillations in the rat OB using 32 channel linear silicon probes. Results presented in (**A**–**D**) were obtained using 3.2 mm electrodes with 100 μm inter-electrode distance. (**A**) Histology illustration based on n = 6 rats OB staining. (**B**) Example raw LFP from 32 channels (y axis). Colorbar scale is in mV. (**C**) Depth profile of 80–180 Hz power from all 32 channels. Power decreases around mitral layer. x-scale is in mV ^2^ (**D**) Phase of 80–130 Hz oscillations which changes phase around mitral layer. Delta changes phase below the mitral. x-scale is in degrees. Results presented in (**E**–**H**) were obtained using 32 channel electrodes with an intercontact distance of 20 μm, and spanning 0.64 mm. Analyses for this data are the same as in (**A**–**D**) (n = 8). Results presented from (**I**–**K**) shows CSD analysis for 20 μm spacing electrode. (**I**) raw example CSD. (**J**) CSD filtered in 0.3–3 Hz band. Plots were then aligned in respect to the histology and averaged across rats n = 8. (**K**) CSD filtered in 80–130 Hz band (**L**) Average correlation plot between of local HFO and MUA density (n = 4). *grn* granule layer, *EPL* extraplexiform layer and *glom.* glomerulur layer.

To determine if 80–130 Hz activity within the OB was localised to particular layers we mapped this rhythm using two types of 32 site linear silicon probes (3.2 mm long with 100 μm spacing and 0.64 mm long with 20 μm spacing) (Fig. 3A,E). 80–130 Hz power was substantially larger more ventrally in the granule layer which was clearly visible in the 3.2 mm probe recordings (Fig. 3B). We observed a sharp reduction in the power of this rhythm close to the mitral layer (Fig. 3C,G). We also observed this phase-shift of activity close to the mitral layer (Fig. 3D,H). For electrodes that did not cross the mitral layer 80–130 Hz oscillations were synchronous across all contacts, on a cycle by cycle basis. Notably, we also found that slow oscillations also reversed phase, but more ventrally within extraplexiform layer (EPL) and glomerular layers (Fig. 3D,H).

Since field potentials reflect neuronal activity from a broad area, we next reconstructed the underlying sources of 80–130 Hz oscillations and the slow oscillation. We used the kCSD method to accurately identify the spatial and temporal profiles of changes in membrane current, underlying the field potential (see "Methods" for computational details). In the raw signal, triggered on 80–130 Hz oscillatory events, we found dipole-like spatiotemporal profiles across the mitral and granule cell layers (Fig. 3I). CSD reconstruction (average for n = 8) was filtered for slow frequencies (0.3–3 Hz) and we observed dipole-like structure that propagates from glomerular layer, before emergence of 80–130 Hz activity, to EPL layer as the 80–130 Hz power rises in time (Fig. 3J). We next filtered the CSD in the 80–130 Hz band and found strong dipoles around the mitral layer (Fig. 3K average across 8 rats). Individual traces from time point zero, from all rats, showing delta and 80–130 Hz dipoles are presented in supplementary Fig. Supplementary S3, respectively. Analysis of the Multi Unit Activity (MUA >500 Hz LFP oscillations) shows that 80–130 Hz oscillations are driven by local spiking of the mitral/tufted cells (Fig. 3L). Correlation computed between local 80–130 Hz activity and envelope of multiunit density (see "Methods") show that spikes does not change phase together with 80–130 Hz—spikes occur in a trough of the oscillation around mitral cell layer.

### Blockade of GABA-A and AMPA but not gap junctions disrupts KX-dependent 80–130 Hz oscillations.

**Figure 4:**
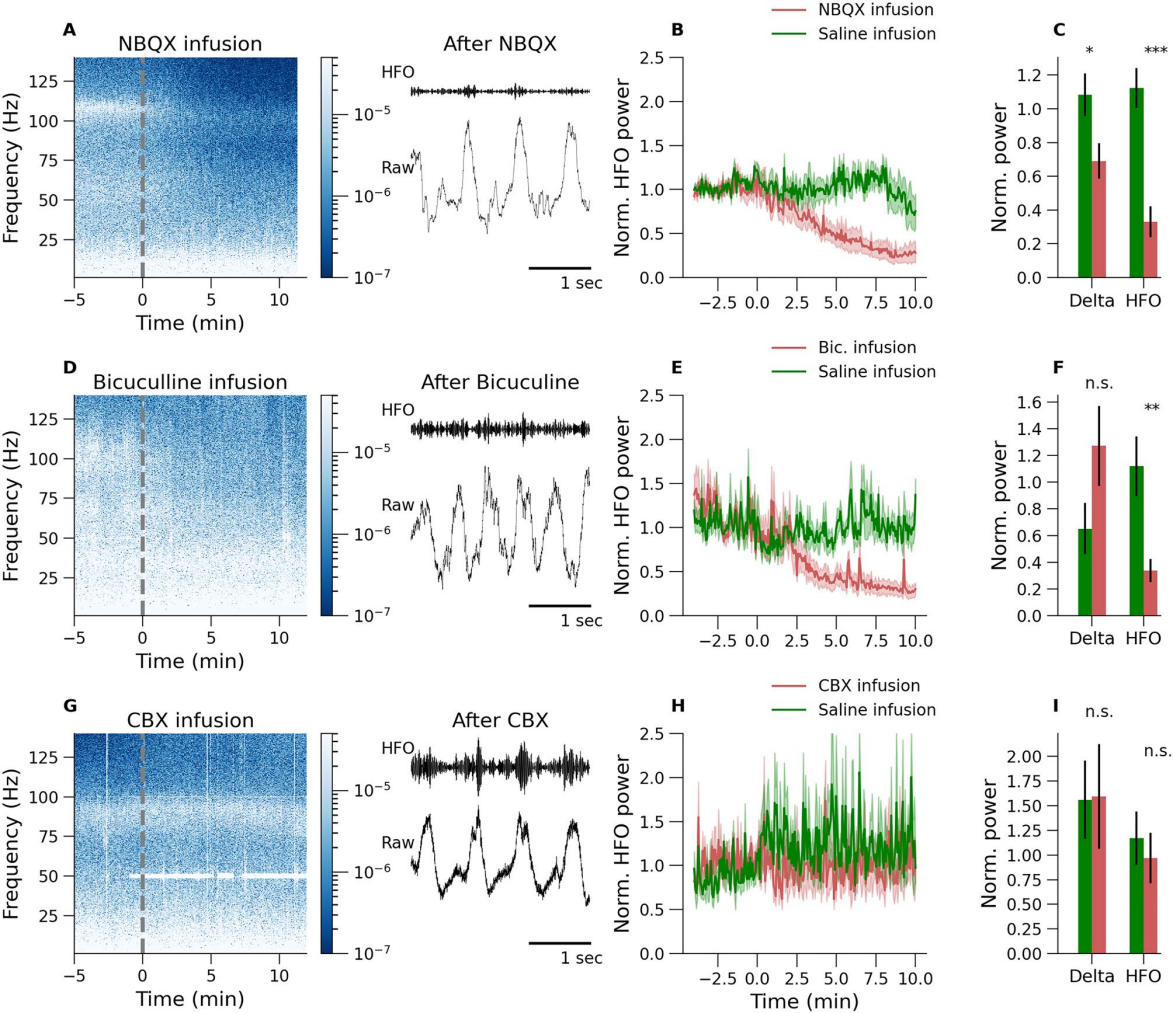
KX-dependent 80–130 Hz activity is AMPA dependent (**A**–**C**) and GABA-A dependent (**D**–**F**) but independent of gap junctions (**G**–**I**). Drugs were infused to unilaterally, and saline infused on the opposite side. Examples of the time courses (dotted line indicates the moment of infusion) and short 3 s data epochs of the raw and 80–130 Hz band pass-filtered waveforms are shown. (**A**–**C**) shows results for NBQX local infusion to the OB which caused a significant reduction in power of the 80–130 Hz oscillations and a smaller but significant effect for delta (**C**, n = 7). GABA-A receptor blockade with bicuculline infusion also reduced 80–130 Hz power without significantly affecting delta power (**D**–**F**, n = 5). Gap junction blockage with carbenoxolone had no significant effect on the 80–130 Hz rhythm, nor delta power (**G**–**I**, n = 4). (*p<0.05, **p<0.01, ***p<0.001 and n.s. (not significant) with respect to saline infusion).

Excitation, inhibition and gap junctions can all underlie mechanisms of fast oscillatory activity in the brain^33^. To unveil the receptor mechanisms generating 80–130 Hz under KX, we carried out a series of pharmacological experiments using unilateral infusion of bicuculline, NBQX, or carbenoxolone (0.5 μl) (Fig. 4). In the presence of KX, the effect of bicuculline was rapid with potent reductions in 80–130 Hz power being visible in many cases during the infusion (saline vs bicuculline, p = 0.0053, one-way ANOVA, n = 5). By contrast, slow delta oscillations in the OB, largely dependent on respiration, remained clear in the raw signal up to 20 min post infusion and the absolute power of delta was not affected by drug infusion (Fig. 4A–F) (saline vs bicuculline, p = 0.098, one-way ANOVA, n = 5). NBQX infusion also reduced 80–130 Hz power (saline vs NBQX, p = 0.00011, one-way ANOVA, n = 7), however, in about half the rats we observed a delayed reduction in the amplitude of slow oscillations but this was well after effects on 80–130 Hz activity occurred (saline vs NBQX, p = 0.012, one-way ANOVA, n = 7). Thus, reductions in 80–130 Hz activity post NBQX were unlikely to be due to reduced sensory input, but rather due to AMPA blockade within the OB circuitry. These findings support a role for both GABA-A and AMPA receptors in the generation of the 80–130 Hz oscillation we recorded. Carbenoxolone did not affect 80–130 Hz activity (saline vs carbenoxolone, p = 0.54, one-way ANOVA, n = 4). Neither delta oscillations were affected (saline vs carbenoxolone, p = 0.96, one-way ANOVA, n = 4) (Fig. 4G–I). Thus, most probably, gap junction connections do not play a role in 80–130 Hz rhythm generation.

### Removal of piriform and surrounding regions do not disrupt KX-dependent 80–130 Hz oscillations.

**Figure 5:**
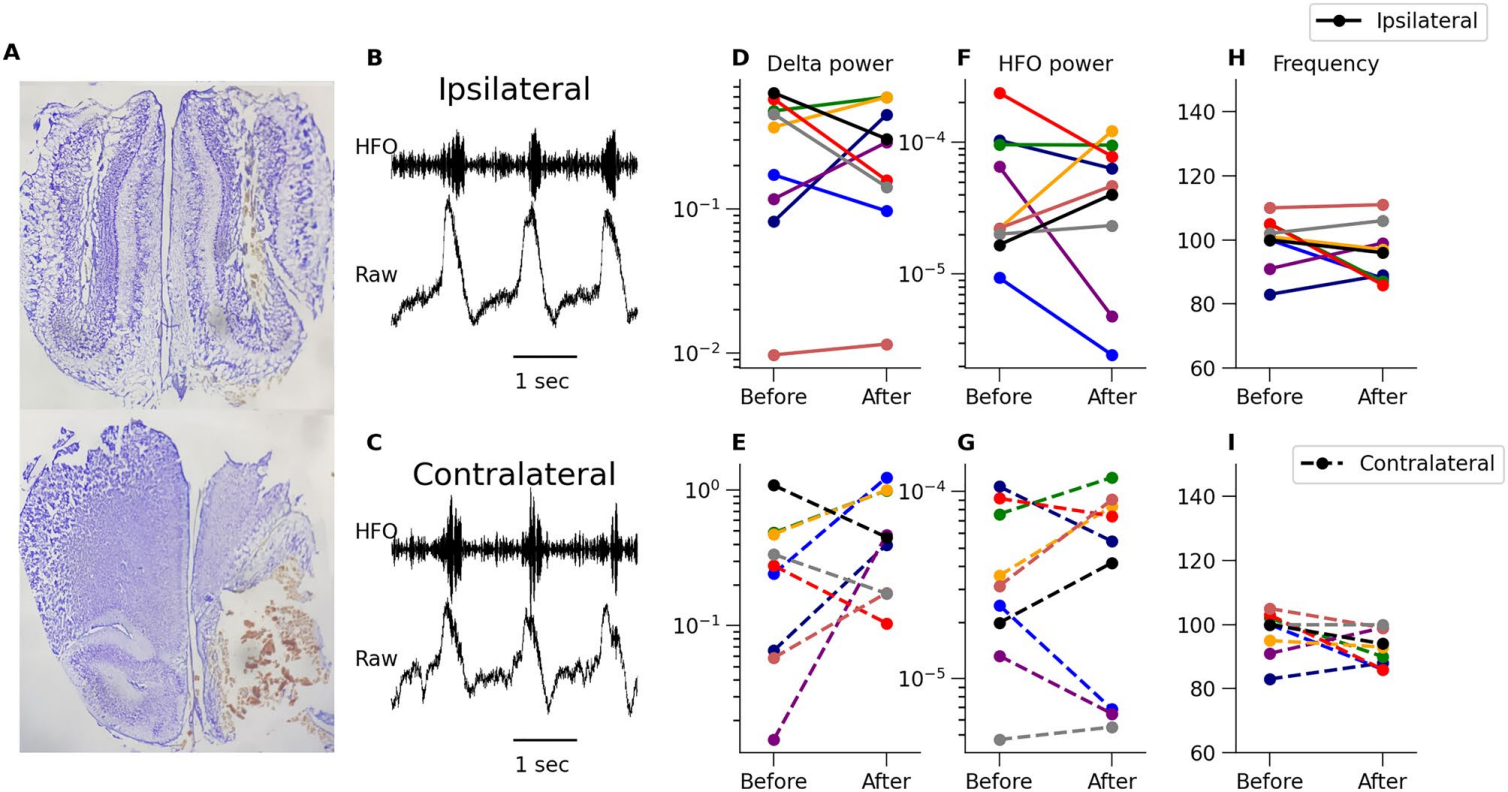
KX-dependent 80–130 Hz oscillations in the rat OB is not dependent on piriform input. (**A**) Example histology of a rat in which we removed the piriform cortex and surrounding areas. (**B**,**C**) Example waveforms on the ipsilateral and contralateral side after the surgical excision, top waveform was filtered in the 80–130 Hz band. (**D**–**I**) We did not observe any significant differences after tissue removal in power of delta (**D** ipsilateral and **E** contralateral side), power of the 80–130 Hz band (**F** ipsilateral and **G** contralateral side) or dominant frequency of the 80–130 Hz rhythm (**H** ipsilateral and **I** contralateral side) (n = 9). Each experimental rat is shown with a different color, the same for ipsilateral and contralateral side.

The piriform cortex is a major downstream structure for OB projections. Pyramidal neurons of the piriform cortex also send dense projection back to OB^38,39^ which have been proposed to control the gain of OB activity^40^. This prompted us to examine the potential role of the piriform cortex, and surrounding regions, on 80–130 Hz activity recorded in the OB. Under KX anesthesia, whilst recording OB LFPs, we gradually removed cortical tissue, starting laterally and moving medially. We did this until the base of the skull was exposed to ensure that a large amount of the piriform cortex has been extracted (Fig. 5A). Example raw LFP and 80–130 Hz (HFO) filtered signals recorded after extraction are presented in Fig. 5B,C. Comparison of LFP oscillations from the dissected vs. intact side had similar power of delta (Fig. 5D,E, p = 0.22 and p = 0.77, both Student’s paired t-test, n = 9) and 80–130 Hz activity (Fig. 5F,G, p = 0.5 (Student’s paired t-test) and p = 0.68 (Wilcoxon test), n = 9). There was also no significant change in the frequency of this rhythm after excision of brain tissue (Fig. 5H,I, p = 0.25 and p = 0.12, both Student’s paired t-test, n = 9). We did not dissect right up to the midline due to the presence of major vessels. However, in four rats the anterior commissure (which carries centrifugal fibers to the OB) and olfactory tubercle was also partially or fully transected, which did not markedly influence oscillatory activity recorded in the OB. We were not able to dissect to the midline due to the presence of the anterior cerebral artery, which is necessary for circulation of blood to the OB and other anterior brain regions. Unexpectedly, on occasions when this vessel was punctured we observed a transient increase in 80–130 Hz activity followed by attenuated activity. We do not currently understand this hemodynamic effect but suspect it is associated with anoxia, and appears in line with another study showing cardiovascular arrest also induced a transient burst of fast oscillatory activity^8^.

### KX-dependent 80–130 Hz oscillations are present in the OB, but not thalamus or visual cortex in cats.

**Figure 6:**
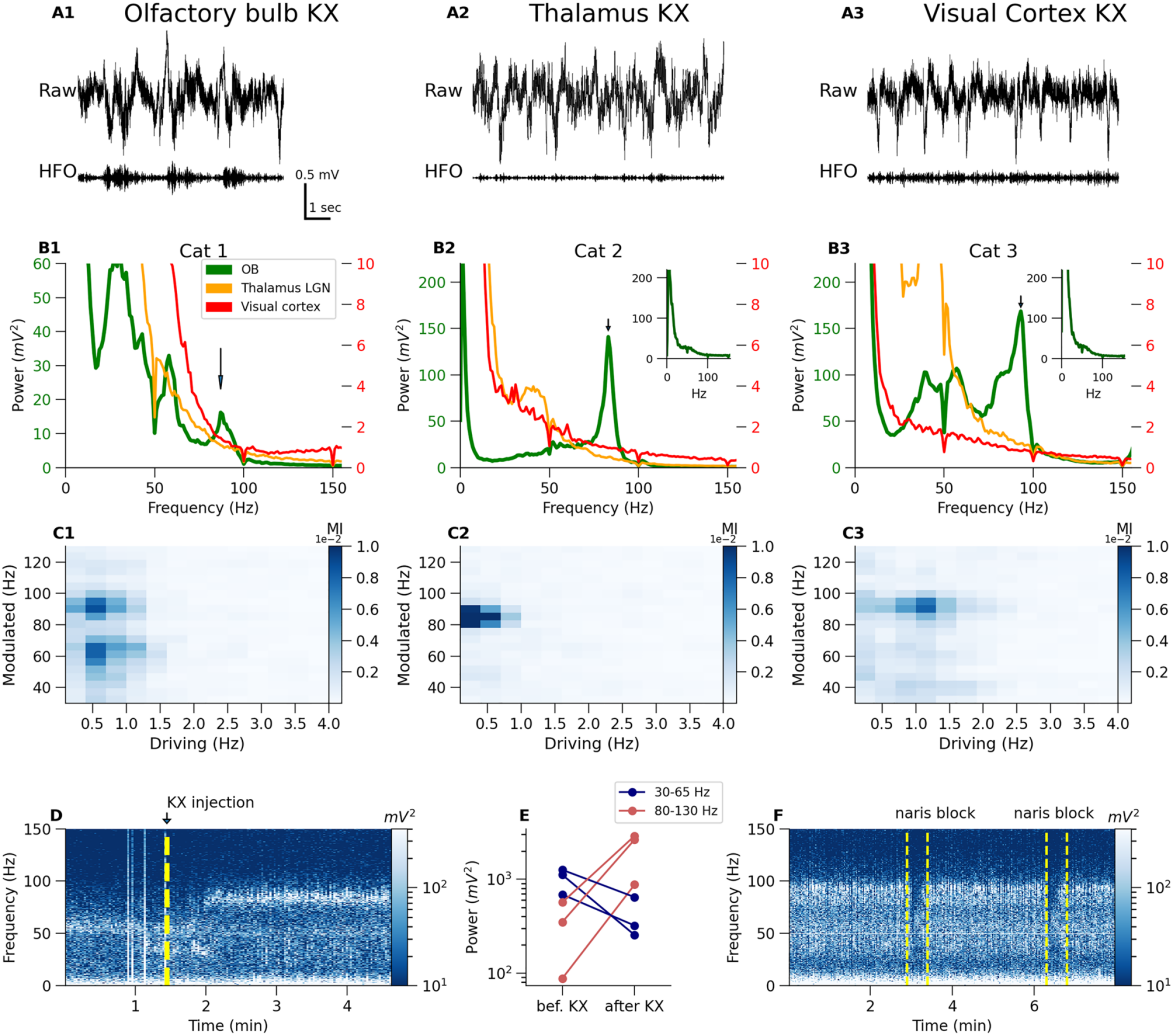
KX-dependent 80–130 Hz oscillations occur in the OB, but not thalamus or visual cortex of cats. (**A**) Example raw waveforms from one cat from three different brain regions. Raw signal is presented in top rows and 80–130 filtered signal is in bottom row. (**B**) Power spectrum from 10 min of recordings. We observed $\sim$ 90 Hz distinct band in OB power spectra of all three cats (indicated on the power spectra by an arrow). In one cat with an electrode in the posterior OB the power of this rhythm was smaller (cat 1). There was no clear peak in the power spectra corresponding to this activity in the thalamus or visual cortex. There is separate y-scale for OB power (green, left) and for thalamus and visual cortex (red, right). Propofol anesthesia attenuated 80–130 Hz activity in the OB (insets, cat 2 and cat 3). (**C**) Modulation index score computed from single OB channels for all three cats. Color strength of the ’pixel’ represents power of the modulation for a given slow (driving frequency) and fast (modulated frequency) oscillation extracted from the raw signal. (**D**,**E**) show the rapid effect on OB oscillatory activity after supplementary infusion of KX, associated with increased power of the 80–130 Hz band with parallel decrease of the power of 30–65 Hz activity. The same phenomena were observed in rats; compare Fig. 1. (**F**) shows the effect of unilateral naris blockade of a cat which was associated with a reduction in 80–130 Hz power.

A fundamental issue when examining drug-related brain activity is whether similar electrophysiological signatures are present in higher mammals. We therefore extended our studies to include cats (n = 3) recording simultaneously from the OB, the lateral geniculate nucleus (LGN) of the thalamus, and from the visual cortex (Fig. 6A1–A3). A $\sim$ 90 Hz oscillation was recorded in the OB of two cats under KX anesthesia (Fig. 6B2–B3) which occurred as discrete bursts (Fig. 6A1). In the cat with weak 90 Hz power (cat 1) histology revealed electrode placement at the edge of the OB which was associated with fast activity of comparable frequency but of much smaller power (Fig. 6B1). For histology see Fig. Supplementary S4. KX fast oscillations were not clearly visible in thalamus or visual cortices, demonstrating certain neuroanataomical selectivity to the OB. In line with our previous findings that ketamine-dependent HFO can be attenuated by various types of anesthesia^29^, we found that propofol (administered at the end of the KX study) also attenuated fast oscillations associated with KX in two cats (Fig. 6B2 and B3 insert).

Modulation index for the OB LFPs of each cat is shown (Fig. 6C1–C3), see "Methods" for computational details. The fast rhythm under KX was coupled to local slow oscillations in OB (strong blue spot around 1 Hz–90 Hz pixel).

KX was infused intravenously at a rate of 0.2 ml every 20 min which provided a window to determine any electrophysiological changes just after administration of KX. Prior to administration, gamma $\sim$ 60 Hz oscillations were present in the OB, then immediately after infusion a clear 90 Hz oscillation was visible (Fig. 6D). This effect was reproducible across several infusions (Fig. 6E) and no clear impact of infusion was seen in the thalamus or visual cortex (Fig. Supplementary S5 A and B). Nasal respiration is known to drive fast and slow oscillations in the OB^41^. To test if the KX rhythm in cats was dependent on nasal airflow we applied short unilateral naris blockade, in two cats, and found this was associated with a reduction in 90 Hz oscillation power (Fig. 6F). In one cat, in addition to the 90 Hz activity (described above) we also observed a strong $\sim$ 160 Hz oscillation (Fig. Supplementary S6 A and B) present exclusively in the bulb, which was not responsive to unilateral naris block Fig. Supplementary S6 C and D).

## Discussion

Here, we show that a 80–130 Hz rhythm emerges under KX anesthesia in LFPs recorded from the OB of rats and cats. Although the OB appears largely capable of generating this rhythm in the absence of input from the piriform cortex, input from the naris did modulate this rhythm and bursts of 80–130 Hz activity were frequently coupled to slow OB ’delta’ oscillations. This is in line with a large body of evidence demonstrating that nasal respiration entrains rhythmic activity in olfactory networks^42,43^. It is also consistent with our recent study showing nasal respiration modulates ketamine-dependent HFO in freely moving rats, although in waking states HFO preferentially coupled with theta (around 7 Hz) frequencies^25,37^. Xylazine alone had no significant impact on 80–130 Hz power indicating dependence of this rhythm on NMDA receptor blockade. Using a 3.2 mm probe we mapped, almost entirely, the dorsal-ventral aspect of the rat OB. The 80–130 Hz rhythm was progressively larger in amplitude in mid-ventral areas of the OB. Axonal projections of mitral/tufted cells are highly spatially localized, whereby axons of cells in the ventral OB travel through ventral regions of the granule layer to the olfactory tract^44,45^. CSD analyses, revealed the presence of two spatially and temporally distinct current dipoles. A sink, around 1 Hz, which followed the breathing cycle, was localized close to the EPL layer and a faster 80–130 Hz microcurrent dipole was found closer to the mitral layer. Current sinks indicate flow of the positive ions (i.e., potassium, calcium) into neurons^46,47^. Stimulation of the olfactory nerve to mimic afferent input, generates a sink in the glomerular and EPL layers^41,48^ and induces slow spontaneous 0.5–5 Hz rhythms^49^ consistent with respiratory drive. We also found that KX mitral/tufted neurons fire in phase with 80–130 Hz, consistent with a previous finding under KX reporting mitral/tufted firing over 100 Hz and associated with inhalation/exhalation transitions^50^. Our previous work in freely moving rats given a subanesthetic dose of ketamine also showed spiking phase locked to a fast 130–180 Hz activity^26^.

The OB receives excitatory and modulatory input from the piriform cortex and centrifugal fibers^38,51^. However, these projections do not appear to make a significant contribution to the 80–130 Hz rhythm since it persisted even when large amounts of the piriform and commissural areas were removed. Notably, slow respiratory rhythms were also preserved confirming the primary drive of this activity was likely from nasal input. These inputs are not essential for gamma oscillations in the OB^41^ indicating 80–130 Hz oscillations, like gamma, are generated within by the intrinsic circuitry of the OB.

Fast oscillations typically require interplay between excitatory and inhibitory transmission^3^. Within the OB fast oscillations can be generated through glutamatergic release by mitral cell dendrites onto granule cell spines and reciprocal GABA release to locally inhibit depolarization spread^52^. In an attempt to look at potential receptor mechanisms within the OB we carried out a series of unilateral microinfusion studies. We showed that the KX 80–130 Hz rhythm was dependent on both inhibitory (GABA-A antagonist; bicuculline) and excitatory components (AMPA antagonist; NBQX). However, gap junctions, which can also underlie both physiological and pathological fast rhythmogenesis^53^ and are expressed by mitral cells^54^, were not implicated in this rhythm.

Although oscillations are often investigated in single isolated bands, important inter-band interactions can underlie rhythmogenesis in the brain. In our rat and cat studies we observed a rapid switch from classical 30–60 Hz gamma to faster 80–130 Hz activity Figs. 1 and 6 D and E. With respect to gamma, Lepousez and LLedo have shown previously that local NMDA blockade in the OB reduces gamma power in mice^35^, but did not report on activity $>100$ Hz activity. There are certain mechanistic similarities between gamma^35^ and the HFO rhythm we recorded in the OB. Both oscillations are reduced by AMPA blockade, and gap junctions blockade was without effect. GABA-A blockade at high doses initially suppresses gamma power followed by a rebound increase in low-gamma power around 30 min later (in our study, we did not record for < 30 min). However, at least in the OB, there appears to be a fundamental distinction in the generation of gamma and ketamine-dependent HFO; gamma (both high and low) requires activation of NMDAR, whereas HFO in the OB requires blockade of these receptors^55^.

Modeling studies have shown that granule cell excitability can control the frequency of OB network oscillations^56^. Under ketamine, reciprocal granule-mitral communication would be suppressed since NMDA receptors are necessary for Ca2+ influx and subsequent GABA release^52,57^. Thus, other excitatory-inhibitory networks would be predicted to underlie the generation of the ketamine-dependent fast rhythm we report here. There is an abundance of other types of inhibitory interneurons within the OB which can shape mitral/tufted cells behavior^58^. How might such fast oscillations be achieved in the OB? Clues may be found in other areas known to generate both gamma and faster oscillations. A good example is the hippocampus, where inhibitory parvalbumin (PV)-positive basket cells generate a ripple frequency. PV positive interneurons have been identified in the EPL layer of the OB, morphologically similar to basket cells^59^, which make reciprocal contacts the perisomatic region of mitral cells^60^. In the OB, as elsewhere in the brain, PV interneurons are fast spiking^61^. In the OB, these cells can fire around 170 Hz, and are modulated by respiration^62^. They are thought to mediate broad lateral inhibition since they receive input and inhibit mitral cells along their long secondary dendrites^63^. Importantly, excitation of PV-positive interneurons by mitral cells is chiefly mediated by AMPA receptors, with only a weak contribution by NMDA receptors^62^. Although PV-positive cells innervate mitral cells more densely than granule cells, very little is known about the capacity of this network to generate oscillations. Given the precedent that these cells can generate fast rhythms (>100 Hz)^62^, we suggest that the movement of air across nasal mechanoreceptors stimulates the olfactory nerve, would depolarizes mitral cells to induce burst firing in PV cells, which generating the reciprocal excitation-inhibition based rhythm recorded here. This network may be relatively weak in the drug-free state (where mitral-granule dendrodendritic interactions prevail), but in the presence of ketamine, reduced inhibitory tone at granule-mitral cell synapses^57^ may permit other networks (e.g. mitral-PV) and associated rhythms to emerge.

Fast oscillations produced by KX (80–130 Hz) and those occurring in freely moving rats after low-dose ketamine (130–180 Hz) shared certain similarities. For example, (1) both oscillations occurred in bursts nested towards the peaks of slower rhythms; (2) both rhythms reversed phase in the vicinity of the mitral layer; (3) nasal airflow drives fast oscillations under KX (shown here) and subanesthetic ketamine in freely moving rats^25^. We also observed certain differences, the frequency was notably slower under KX anesthesia. Although anesthesia tends to attenuate most activity $>40$ Hz there are some examples of ripple frequencies recorded under anesthesia; for example, Ylinen reported that under urethane and ketamine anesthesia the frequency of ripples was slower (100–120 Hz) than in the awake rat (180–200 Hz)^32^. Additionally, Neville and Haberly also reported that the frequency of discrete gamma and beta oscillatory bands is slower under urethane that the corresponding oscillations in awake rats^41^. Finally, we have shown previously, the frequency of ketamine-dependent HFO can drop by as much as 80 Hz after antipsychotic injection^64^, thus this band is highly dynamic and capable of relatively large frequency fluctuations. We also found the power was weaker under KX, versus the subanethetic doses of ketamine in the awake state, which is almost certainly arousal-related and associated with changes in functional brain connectivity under anesthesia.

To our knowledge, almost all previous reports of ketamine-dependent HFO have focused on freely moving rodents^65^. These studies (and also the current paper) show this type of HFO is visible as a clear bump in the power spectra. There is some evidence broadband HFO can be recorded in magnetoencephalography recordings in humans after subanesthetic ketamine^66^. Our finding, that under KX anesthesia, similar fast rhythms occurred in both rats and cats, suggests that this activity may be conserved in other higher-order mammals. However, further studies are warranted to confirm if this is a fundamental effect of ketamine on mammalian networks, and potential relevance to the psychotomimetic and antidepressant properties of this drug.

## Methods

### Surgery and chronic recordings

All experiments were conducted in accordance with the European community guidelines on the Care and Use of Laboratory Animals (2010/63/EU) and approved by the 1st Local Ethics Committee for Animal Experiments in Warsaw, Poland. Thirty male Wistar rats (250–350 g) were used in this study. In 8 rats, tungsten electrodes (125 μm, Science Products, Germany) were implanted bilaterally in OB (AP+7.5, ML = +0.5, DV = 3–3.5 mm). LFP recordings were carried out before and after injection intraperitoneal of ketamine 25 mg/kg, and anesthetic doses of ketamine 100 mg/kg + xylazine 10 mg/kg (KX), and ketamine 200 mg/kg with 3–4 days separating each recording session. Under KX anesthesia the left or right naris was occluded using a soft piece of silicon rubber to block nasal respiration. At the end of the experiments the rats were killed by a lethal dose of pentobarbital. The brains were post-fixed in 4% paraformaldehyde solution. Brains were dissected and placed in a 10% followed by 30% sucrose solution for 2–4 days. Electrode locations were determined on 40 μm Cresyl violet (Sigma, UK) or Hoechst (Sigma, UK) stained sections.

### Thermocouple and LFP recordings

For acute studies, eight rats were initially anesthetized using isoflurane during which time electrodes were implanted in the OB and thermocouples in the frontal recess of the naris on the ipsilateral side. When electrodes were in place isoflurane anesthesia was replaced with KX by gradual injection of the cocktail and removal of isoflurane. Initial isoflurane exposure was necessary due to well-documented variable surgical plane anesthetic responses in rats compared to KX alone^67^.

### Silicon probe recordings

A total of 14 rats were used for spatial mapping of oscillatory activity in the rodent OB. Rats were prepared for acute recordings as described above. Recordings were carried out in the OB using 32-channel silicon probes Edge-10mm–100–177 (n = 6 rats, Neuronexus) and Edge-10mm–20–177 (n = 8 rats). The electrodes were separated by an interelectrode distance of 100 (long probe) and 20 μm (short probe). Prior to recording electrodes were dipped in a 5% solution of DIi (Sigma) dissolved in DMSO (Sigma). The track of the electrode was visualized using a fluorescent microscope.

### Local drug infusion

Rats were initially anesthetized using isoflurane and implanted with a guide-electrode complex in the left and right OB. Following implantation, isoflurane was substituted for KX anesthesia (see above for further information). LFPs were recorded bilaterally and when a stable fast oscillation was visible (80–130 Hz). NBQX (2 μg, Sigma, n = 7), Bicuculline methiodide (0.05 μg, Sigma, n = 5) and Carbenoxolone disodium salt (1 μg, Sigma, n = 4) were infused into the bulb. Saline was infused to the opposite bulb. For infusion, internal cannulae (28 gauge, Bilaney) that extended 2 mm below the tip of the guides were inserted for 60 s followed by 60 s infusion of NBQX, bicuculline, CBX or saline (volume 0.5 μl). Rats were recorded for up to 30 min post infusion.

### Dissection of brain tissue

Rats were initially anesthetized by isoflurane for implantation of electrodes in the left and right OB. Following removal of the overlying skull isoflurane anesthesia was replaced by KX. Under stable KX anesthesia we drilled the perimeter of a large cranial window approx. 7 mm × 7 mm (left hemisphere) from the midline to the lateral edge of the skull and removed the overlying bone. The exposed brain was dissected (using aspiration and a scalpel) in a lateral-medial direction until the base of the skull had been reached. LFPs from the left and right OB were recorded prior to and immediately after dissection.

### Cat experiments

All experiments were conducted in accordance with the European community guidelines on the Care and Use of Laboratory Animals (86/609/EEC) and approved by the 1st Local Ethics Committee for Animal Experiments in Warsaw, Poland. Three healthy, mature cats (one female, two male) were used. Cats were initially anesthetized using a bolus KX i.p. injection . An intravenous catheter was implanted in a saphenous vein for supplementary KX administration and 0.1–0.2 ml was administered every 20 min. Cat’s were placed in a stereotaxic frame and electrodes (32-channel silicon probes or in-house 16-channel electrodes made of 25 mm tungsten wire) implanted in the OB (AP approx. +30 mm from the interaural line, ML = 2–4 mm, DV = 3.4 mm from surface), and also in the thalamus (AP +6.5 mm, ML = 9 mm, DV = 13.5 mm) and visual cortex (AP = −6 to −9 mm, ML = 3–9 mm, DV = 1–2 mm). Thalamic and cortical electrodes were implanted as part of a separate study examining visual-evoked activity which was carried out after the KX experiment. Following acquisition of electrophysiological data the naris was briefly closed for 10 s (in two cats), anesthesia was then replaced with propofol for vision experiments (not presented here).

### Data analysis

Recorded signals were processed using SciPy signal and NumPy Python libraries. Analysis included bandpass filtering using Butterworth filters. Power of dominant frequency and dominant frequency were evaluated using Welch transform from 60 s windows. To establish phase relation in KX HFO we first used Hilbert transform to find a maximum activity of HFO burst and then computed shift in time relative to peak of delta oscillations (score is rescaled to radians). Several hundred HFO bursts were used to compute intertrial phase clustering (ITCP) defined as $ITPC \,=\, |n^{-1}\sum _{r=1}^{n}e^{ik}|$, where *k* is the relative phase of the burst and *n* is the number of trial.

To study correlation of thermocouple’s rhythm and LFP signal oscillation we filtered the signal in delta frequencies 0.3–3 Hz and KX HFO 80–130 Hz. We computed a Pearson correlation score between the delta band of thermocouple and the envelope of HFO signal computed with Hilbert transform. To confirm our hypothesis that HFO is modulated by breathing rhythm we used comodulogram analysis for the two signals. Comodulogram matrix was computed using open-source Python library described in^68^. We used the “Tort” method from pactools Python package, which seems to find a compromise for proper resolution in “phase” and “amplitude” signal and is based on classic phase–amplitude coupling method. We evaluated statistical significance of the coupling using resampling test of MI martices between groups of rats that were under KX and isoflurane anesthesia.

For multielectrode recordings, KX-HFOs significant bursts were detected using 3 standard deviation threshold from top (short silicon probe) and middle (long silicon probe) channels used as a reference. We computed phase shift between channels using maximum correlation score in respect to reference channel and averaged the score across rats. CSDs were reconstructed using kCSD algorithm method from^69,70^ and available at https://github.com/Neuroinflab/kCSD-python. We reconstructed CSD first and then filtered spatio-temporal CSD picture in delta 0.3–3 Hz and KX-HFO 80–130 Hz frequency bands. For multiunit activity analysis we first filtered the signal above 500 Hz for every HFO burst/event. Then we extracted candidates for spikes with 3 standard deviation criterion and represented them as discrete events in time. As a final step we made a histogram from aggregated (across HFO events) spikes and computed Pearson correlation coefficient between spike histogram and average HFO waveform. We repeated this kind of analysis for all channels independently.

Shaded regions in all plots and whiskers of the bar plots represent standard deviation of the mean (s.e.m). All sample groups were tested with Shapiro-Wilk’s test for normality. If the data was normally distributed we used a one-way ANOVA test for independent experiments or paired Student t-test for repeated measures. If the score of the normality test was below p = 0.05, we used the Wilcoxon test or Friedman test followed by Nemenyi post-hoc testing. We used tests from SciPy Python library. Additionally, we used a resampling test (100 000 draws with return) to compare modulation index matrices for KX and isoflurane anesthetized rats (n = 8). All figures were made using Matplotlib Python library^71^.

## Supplementary Information

Supplementary Information.
