# Supplementary material for: Network and synaptic mechanisms underlying high frequency oscillations in the rat and cat olfactory bulb under ketamine-xylazine anesthesia
Source: Sci Rep. 2021 Mar 18;11:6390. doi: 10.1038/s41598-021-85705-5 (PMC7973548; doi:10.1038/s41598-021-85705-5)
Supplement: Supplementary file 1 — Supplementary Information. [file 41598_2021_85705_MOESM1_ESM.pdf]

Network and synaptic mechanisms underlying high frequency oscillations in the rat and cat olfactory bulb under ketamine-xylazine anesthesia.

Władysław Średniawa[1,2], Jacek Wróbel[1], Ewa Kublik[1], Daniel Krzysztof Wójcik[1,3], Miles Adrian Whittington[4], and Mark Jeremy Hunt[1,4*]

[1]Nencki Institute of Experimental Biology of Polish Academy of Sciences, 3 Pasteur Street, 02-093 Warsaw, Poland
[2]University of Warsaw, Faculty of Biology, Miecznikowa 1, 02-096, Warsaw, Poland
[3]Jagiellonian University, Faculty of Management and Social Communication, Jagiellonian University, 30-348 Cracow, Poland
[4]University of York, Heslington, York, YO10 5DD, United Kingdom
[*]Correspondence: Mark Jeremy Hunt, Nencki Institute of Experimental Biology, 3 Pasteur Street, 02-093 Warsaw, Poland, m.hunt@nencki.edu.pl

ABSTRACT

Wake-related ketamine-dependent high frequency oscillations (HFO) can be recorded in local field potentials (LFP) from cortical and subcortical regions in rodents. The mechanisms underlying their generation and occurrence in higher mammals are unclear. Unfortunately, anesthetic doses of pure ketamine attenuate HFO, which has precluded their investigation under anesthesia. Here, we show ketamine-xylazine (KX) anesthesia is associated with a prominent 80–130 Hz rhythm in the olfactory bulb (OB) of rats, whereas 30–65 Hz gamma power is diminished. Simultaneous LFP and thermocouple recordings revealed the 80–130 Hz rhythm was dependent on nasal respiration. This rhythm persisted despite surgical excision of the piriform cortex. Silicon probes spanning the dorsoventral aspect of the OB revealed this rhythm was strongest in ventral areas and associated with microcurrent sources about the mitral layer. Pharmacological microinfusion studies revealed dependency on excitatory-inhibitory synaptic activity, but not gap junctions. Finally, a similar rhythm occurred in the OB of KX-anesthetized cats, which shared key features with our rodent studies. We conclude that the activity we report here is driven by nasal airflow, local excitatory-inhibitory interactions, and conserved in higher mammals. Additionally, KX anesthesia is a convenient model to investigate further the mechanisms underlying wake-related ketamine-dependent HFO.

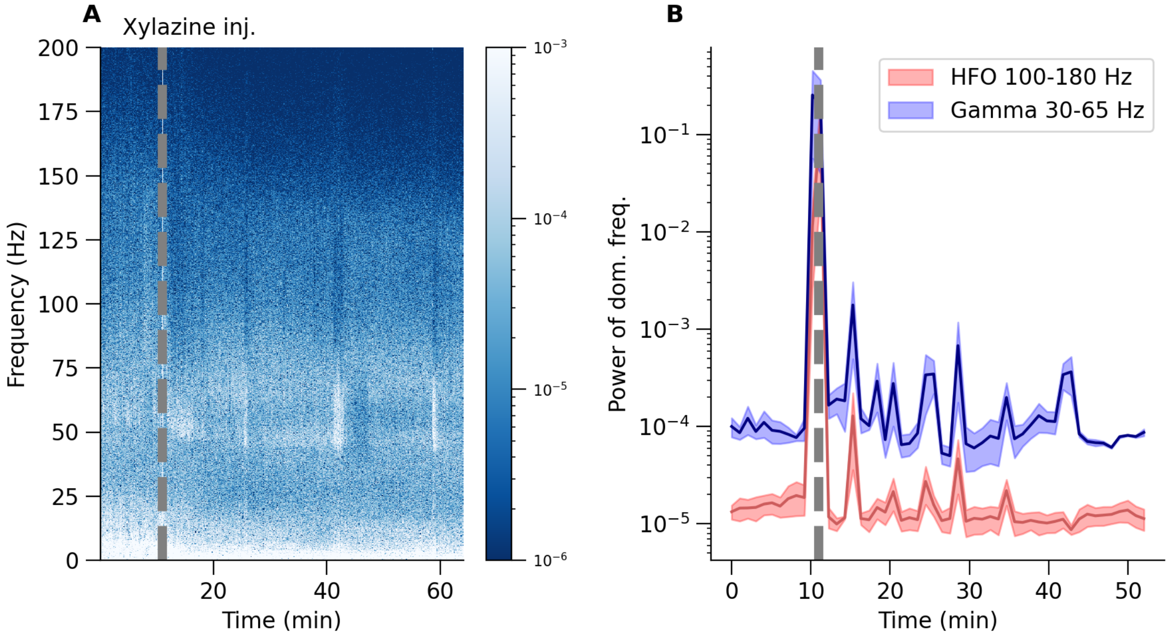

Figure Supplementary S1. A: Spectrogram of the example rat after xylazine injection. B: Extraction of the power of dominant frequency for 30-65 and 100–180 Hz bands. We did not see any change in the power of these bands after xylazine application alone.

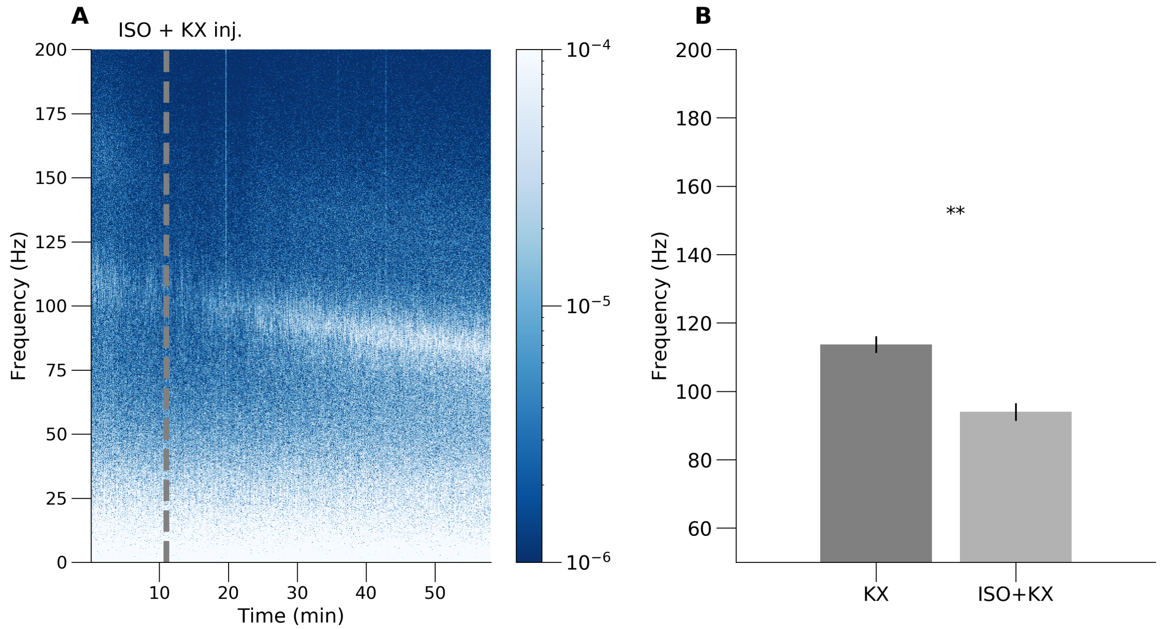

Figure Supplementary S2. A: Example spectrogram of the chronically implanted rat that was exposed first to isoflurane and then got KX injection. B: Analysis of the frequency reduction in rats that were shortly exposed to isoflurane before KX injection.

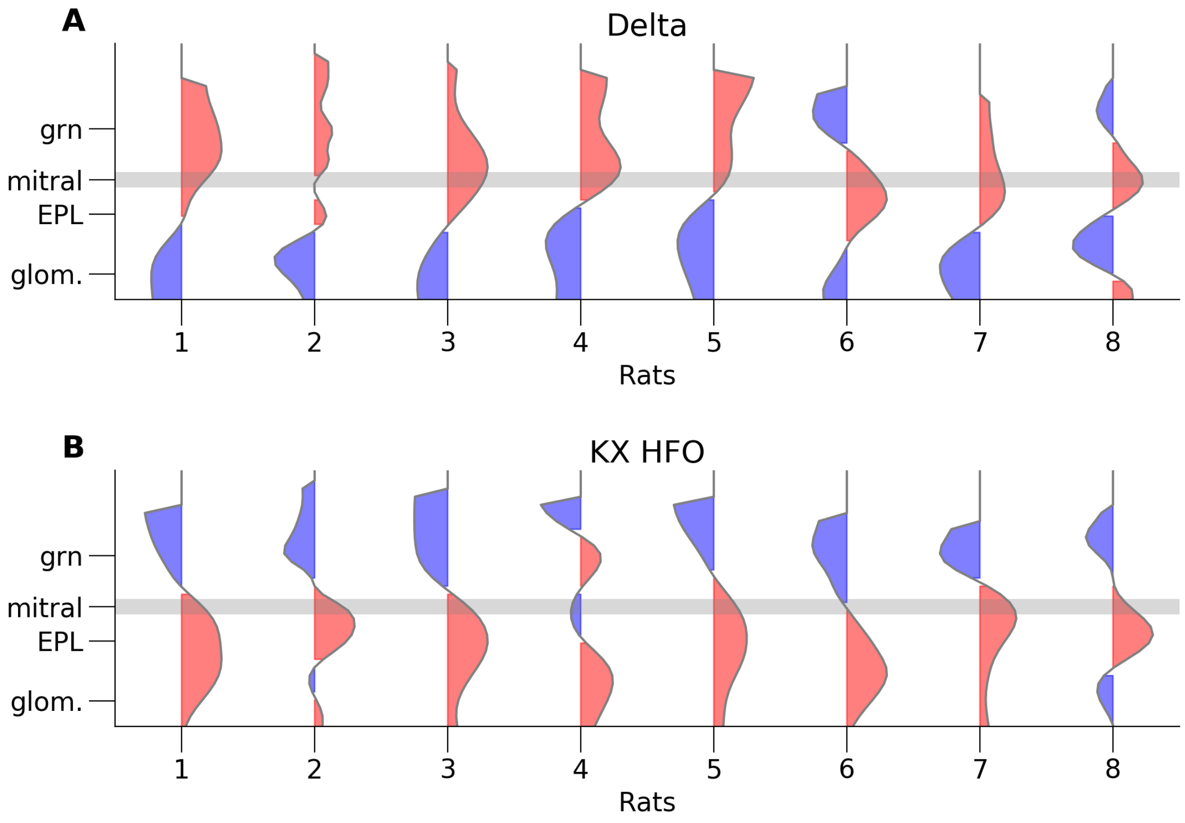

Figure Supplementary S3. CSD profiles from individual rats 1 to 8 extracted from timepoint zero for delta (A) and the KX 80-130 Hz band (B) filtered CSD reconstructions

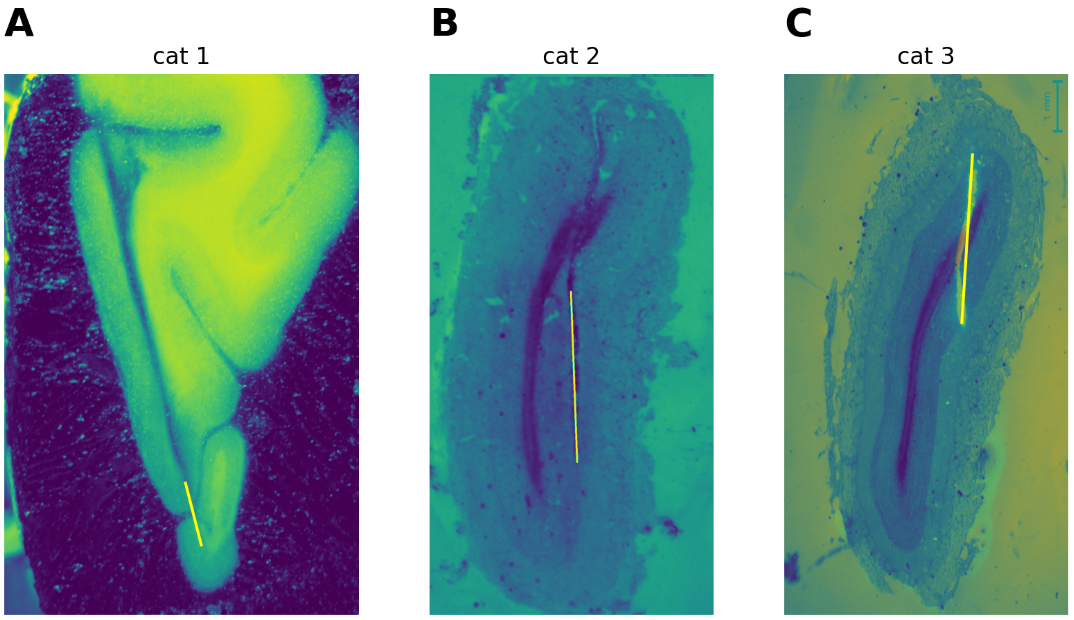

Figure Supplementary S4. OB histology from cat experiments (n=3). Yellow lines represent the position of the electrodes.

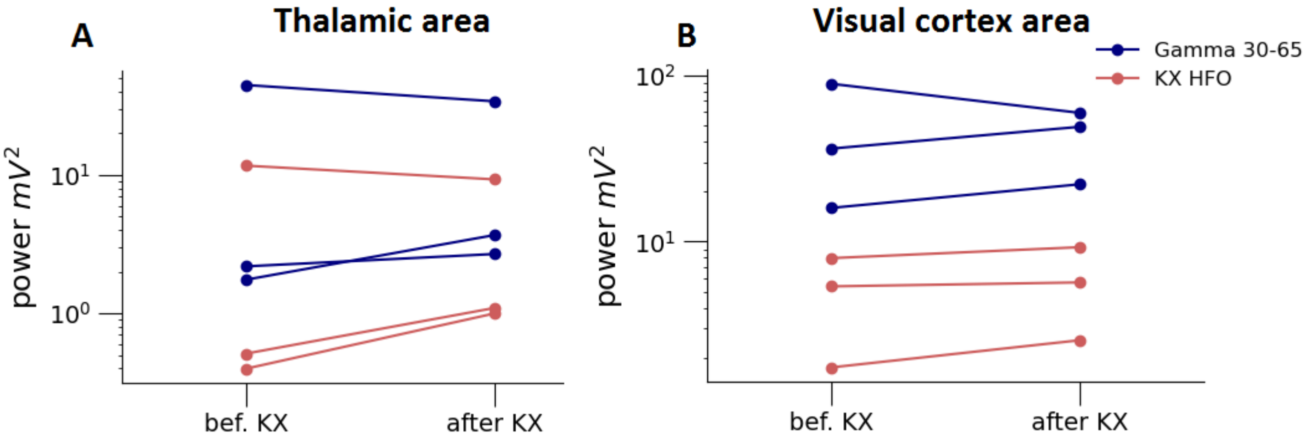

Figure Supplementary S5. Power of the 80-130 band and 30-65 Hz before and after supplmentary KX infusion from thalamus (A) and visual cortex (B). Individual lines represents 3 cats.

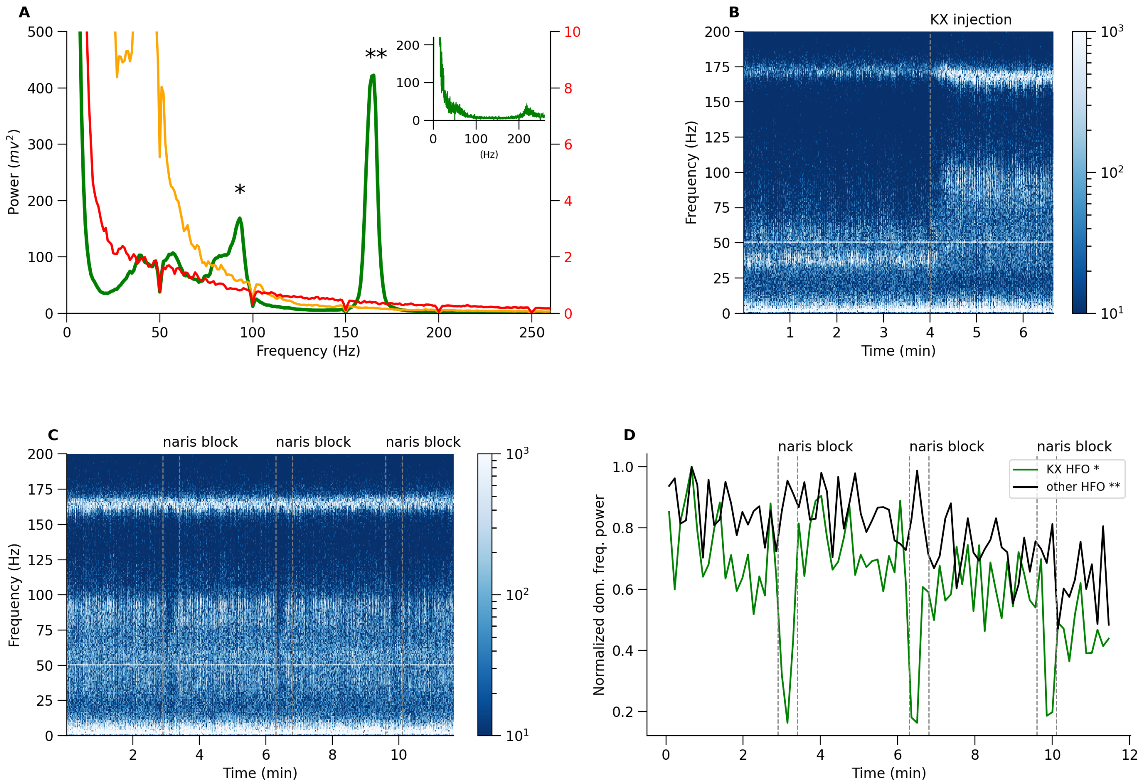

Figure Supplementary S6. A: Power spectrum of the third cat recorded in OB, thalamus and visual cortex. * and ** refers to the first and the second high frequency peaks, respectively. B: Spectrogram of the response to the renewal of KX anesthesia. C: Spectrogram of the cat under KX anesthesia and naris block experiment. D: Extraction of the power of dominant frequency from 80–130 Hz and 150–170 Hz bands during naris block experiment. Note that 160 Hz oscillation is not respond to perturbation in respiration.
